# Methyl 11-hy­droxy-9-[1-(4-meth­oxy­phen­yl)-4-oxo-3-phenyl­azetidin-2-yl]-18-oxo-10-oxa-2-aza­penta­cyclo­[9.7.0.0^1,8^.0^2,6^.0^12,17^]octa­deca-12(17),13,15-triene-8-carboxyl­ate

**DOI:** 10.1107/S1600536813004789

**Published:** 2013-02-23

**Authors:** Sivasubramanian Suhitha, Thothadri Srinivasan, Raju Rajesh, Raghavachary Raghunathan, Devadasan Velmurugan

**Affiliations:** a Centre of Advanced Study in Crystallography and Biophysics, University of Madras, Guindy Campus, Chennai 600 025, India; b Department of Organic Chemistry, University of Madras, Guindy Campus, Chennai 600 025, India

## Abstract

In the title compound, C_34_H_32_N_2_O_7_, the furan ring adopts a twist conformation and both the pyrrolidine rings adopt envelope conformations with O and C as flap atoms. The β-lactam ring makes a dihedral angles of 80.20 (10)° with the furan ring, of 75.55 (10)° with the pyrrolidine ring, of 12.26 (10)° with the meth­oxy­phenyl ring and of 73.77 (13)° with the phenyl ring. The O atom attached to the β-lactam ring deviates by 0.0385 (13) Å from the ring plane. The mol­ecular conformation is stabilized by intra­molecular O—H⋯N and C—H⋯O hydrogen bonds. The packing of the crystal is stabilized by inter­molecular C—H⋯O hydrogen bonds, which form a chain running along the *b* axis.

## Related literature

For general background to β-lactams, see: Banik & Becker (2000 ▶); Brakhage (1998 ▶). For a related structure, see: Sundaramoorthy *et al.* (2012 ▶).
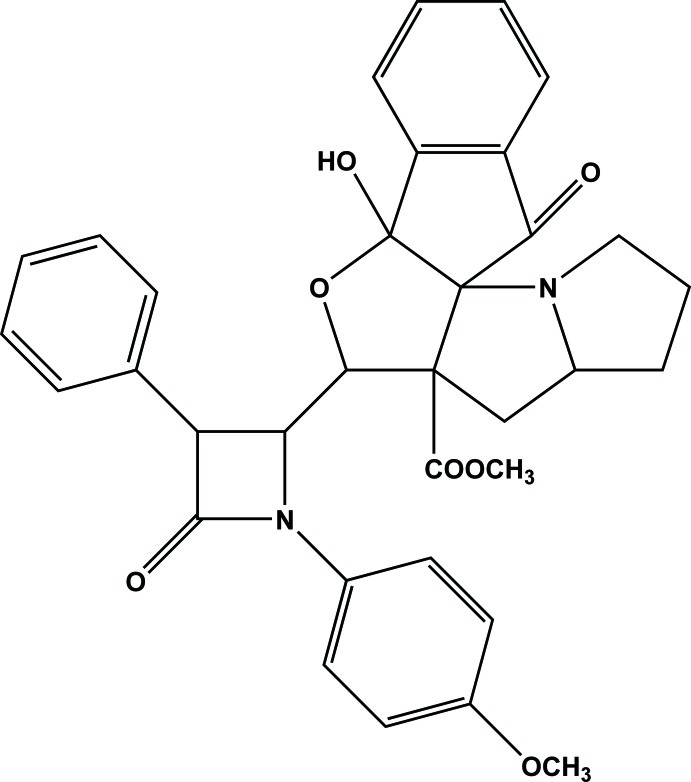


## Experimental

### 

#### Crystal data

C_34_H_32_N_2_O_7_
*M* *_r_* = 580.62
Monoclinic, 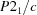

*a* = 10.9030 (5) Å
*b* = 11.8792 (5) Å
*c* = 22.4457 (10) Å
β = 93.963 (3)°
*V* = 2900.2 (2) Å^3^
*Z* = 4
Mo *K*α radiation
μ = 0.09 mm^−1^
*T* = 293 K
0.30 × 0.25 × 0.20 mm

#### Data collection

Bruker SMART APEXII area-detector diffractometer
Absorption correction: multi-scan (*SADABS*; Bruker, 2008 ▶) *T*_min_ = 0.973, *T*_max_ = 0.982
28525 measured reflections
7293 independent reflections
4952 reflections with *I* > 2σ(*I*)
*R*_int_ = 0.029

#### Refinement

*R*[*F* ^2^ > 2σ(*F* ^2^)] = 0.047
*wR*(*F* ^2^) = 0.127
*S* = 1.02
7293 reflections
394 parameters
H atoms treated by a mixture of independent and constrained refinement
Δρ_max_ = 0.20 e Å^−3^
Δρ_min_ = −0.21 e Å^−3^

### 

Data collection: *APEX2* (Bruker, 2008 ▶); cell refinement: *SAINT* (Bruker, 2008 ▶); data reduction: *SAINT*; program(s) used to solve structure: *SHELXS97* (Sheldrick, 2008 ▶); program(s) used to refine structure: *SHELXL97* (Sheldrick, 2008 ▶); molecular graphics: *ORTEP-3 for Windows* (Farrugia, 2012 ▶); software used to prepare material for publication: *SHELXL97* and *PLATON* (Spek, 2009 ▶).

## Supplementary Material

Crystal structure: contains datablock(s) global, I. DOI: 10.1107/S1600536813004789/bt6889sup1.cif

Structure factors: contains datablock(s) I. DOI: 10.1107/S1600536813004789/bt6889Isup2.hkl

Supplementary material file. DOI: 10.1107/S1600536813004789/bt6889Isup3.cml

Additional supplementary materials:  crystallographic information; 3D view; checkCIF report

## Figures and Tables

**Table 1:** Hydrogen-bond geometry (Å, °)

*D*—H⋯*A*	*D*—H	H⋯*A*	*D*⋯*A*	*D*—H⋯*A*
O4—H4*A*⋯N2	0.91 (2)	1.95 (2)	2.6120 (16)	127.8 (19)
C6—H6⋯O1	0.93	2.51	3.122 (2)	124
C8—H8⋯O1^i^	0.98	2.52	3.4047 (19)	151
C28—H28*A*⋯O4^ii^	0.97	2.58	3.5069 (19)	161

Symmetry codes: (i) 
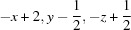
; (ii) 
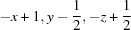
.

**Fig. 1.**
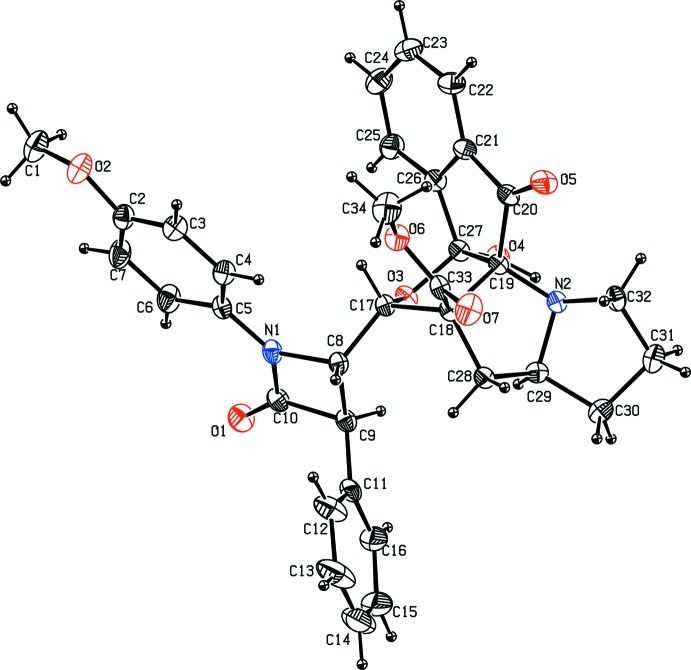
The molecular structure of the title compound, showing displacement ellipsoids drawn at the 30% probability level. H atoms are presented as a small spheres of arbitrary radius.

**Fig. 2.**
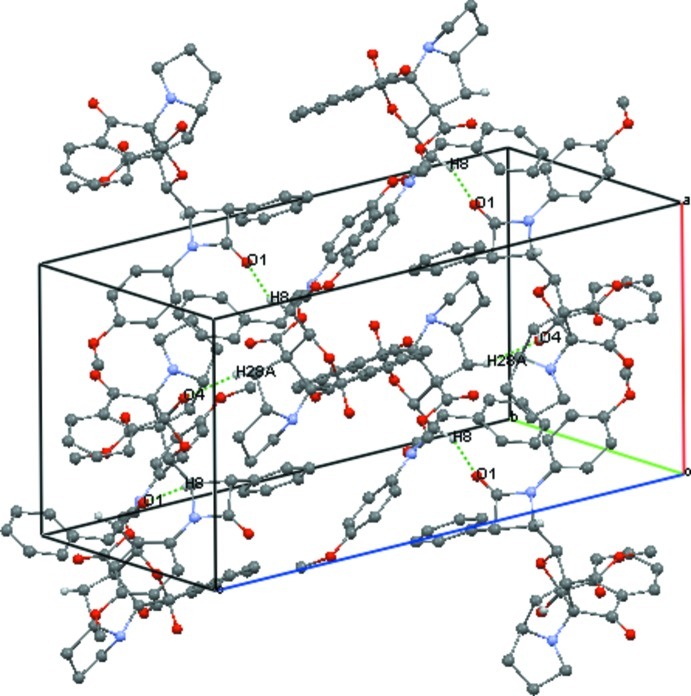
The crystal packing of the title compound viewed down b axis. H-atoms not involved in H-bonds have been excluded for clarity.

## supplementary crystallographic information

### Comment

The role of β-lactam antibiotics is well known (Banik & Becker, 2000). The most commonly used β-lactam antibiotics for the therapy of infectious diseases are penicillin and cephalosporin (Brakhage, 1998). In view of potential applications, the crystal structure determination of the titled β-lactam derivative was carried out. In the title compound (Fig. 1), the β lactam ring makes a dihedral angle of 80.20 (10)° with the furan ring (C17/C18/C19/C27/O3) and a dihedral angle of 75.55 (10)° with the pyrrolidine ring (C18/C19/C28/C29/N2). The β lactam ring makes a dihedral angle of 12.26 (10)° with the methoxy phenyl ring and a dihedral angle of 73.77 (13)° with unsubstituted phenyl ring.

Both the pyrrolidine rings adopt an *envelope* conformation and the furan ring adopts a *twist* conformation. The furan ring makes a dihedral angle of 81.29 (8)° with the pyrrolidine ring, a dihedral angle of 72.61 (9)° with the other pyrrolidine ring(N2/C29/C30/C31/C32). The furan ring makes a dihedral angle of 72.26 (8)° with the cyclopentane ring(C19/C20/C21/C26/C27) system. The oxygen atom (O1) attached with the β lactam ring deviates by 0.0385 (13)Å from the ring plane. The hydroxyl oxygen atom (O4) attached with the furan ring deviates by -0.6644 (11)Å from the ring plane. The oxygen atom (O5) attached to the cyclopentane ring deviates by 0.2042 (13)Å from the ring plane. The molecular conformation is stabilized by an intramolecular O-H···N and C-H···O hydrogen bonds. The packing of the crystal is stabilized by intermolecular C—H···O hydrogen bonds (Fig. 2).

### Experimental

A mixture of methyl 2-(hydroxy(1-(4-methoxyphenyl)-4-oxo-3-phenylazetidin-2-yl) methyl)acrylate (1.0 equiv.), ninhydrin (1.1 equiv.) and proline (1.1 equiv.) was refluxed in methanol. Completion of the reaction was evidenced by TLC analysis. After completion of the reaction the solvent was evaporated under reduced pressure. The reaction mixture was dissolved in dichloromethane and with water followed by brine solution. The organic layer was separated and evaporated under reduced pressure. The crude mixture was purified by column chromatography using ethyl acetate and hexane as eluent (3: 7). The product was dissolved in chloroform and heated for two minutes. The resulting solution was subjected to crystallization by slow evaporation of the solvent for 48 hours resulting in the formation of single crystals.

### Refinement

The hydrogen atoms bonded to carbon atoms were placed in calculated positions with C—H = 0.93 Å to 0.97 Å. They were refined using a riding model with fixed isotropic displacement parameters: *U*_iso_(H) = 1.5*U*_eq_(C) for methyl groups and *U*_iso_(H) = 1.2*U*_eq_(C) for other H atoms. The hydroxyl H atom was freely refined.

### Crystal data

**Table:**

C_34_H_32_N_2_O_7_	*F*(000) = 1224
*M**_r_* = 580.62	*D*_x_ = 1.330 Mg m^−^^3^
Monoclinic, *P*2_1_/*c*	Mo *K*α radiation, λ = 0.71073 Å
Hall symbol: -P 2ybc	Cell parameters from 7293 reflections
*a* = 10.9030 (5) Å	θ = 1.8–28.5°
*b* = 11.8792 (5) Å	µ = 0.09 mm^−^^1^
*c* = 22.4457 (10) Å	*T* = 293 K
β = 93.963 (3)°	Block, colourless
*V* = 2900.2 (2) Å^3^	0.30 × 0.25 × 0.20 mm
*Z* = 4	

### Data collection

**Table:**

Bruker SMART APEXII area-detector diffractometer	7293 independent reflections
Radiation source: fine-focus sealed tube	4952 reflections with *I* > 2σ(*I*)
Graphite monochromator	*R*_int_ = 0.029
ω and φ scans	θ_max_ = 28.5°, θ_min_ = 1.8°
Absorption correction: multi-scan (*SADABS*; Bruker, 2008)	*h* = −14→14
*T*_min_ = 0.973, *T*_max_ = 0.982	*k* = −15→13
28525 measured reflections	*l* = −29→30

### Refinement

**Table:**

Refinement on *F*^2^	Primary atom site location: structure-invariant direct methods
Least-squares matrix: full	Secondary atom site location: difference Fourier map
*R*[*F*^2^ > 2σ(*F*^2^)] = 0.047	Hydrogen site location: inferred from neighbouring sites
*wR*(*F*^2^) = 0.127	H atoms treated by a mixture of independent and constrained refinement
*S* = 1.02	*w* = 1/[σ^2^(*F*_o_^2^) + (0.0518*P*)^2^ + 0.680*P*] where *P* = (*F*_o_^2^ + 2*F*_c_^2^)/3
7293 reflections	(Δ/σ)_max_ < 0.001
394 parameters	Δρ_max_ = 0.20 e Å^−^^3^
0 restraints	Δρ_min_ = −0.21 e Å^−^^3^

### Special details

**Table:**

Geometry. All esds (except the esd in the dihedral angle between two l.s. planes) are estimated using the full covariance matrix. The cell esds are taken into account individually in the estimation of esds in distances, angles and torsion angles; correlations between esds in cell parameters are only used when they are defined by crystal symmetry. An approximate (isotropic) treatment of cell esds is used for estimating esds involving l.s. planes.
Refinement. Refinement of F^2^ against ALL reflections. The weighted R-factor wR and goodness of fit S are based on F^2^, conventional R-factors R are based on F, with F set to zero for negative F^2^. The threshold expression of F^2^ > 2sigma(F^2^) is used only for calculating R-factors(gt) etc. and is not relevant to the choice of reflections for refinement. R-factors based on F^2^ are statistically about twice as large as those based on F, and R- factors based on ALL data will be even larger.

### Fractional atomic coordinates and isotropic or equivalent isotropic displacement parameters (Å^2^)

**Table:**

	*x*	*y*	*z*	*U*_iso_*/*U*_eq_	
C1	1.2979 (2)	0.34508 (19)	−0.00464 (11)	0.0799 (6)	
H1A	1.2334	0.3996	−0.0112	0.120*	
H1B	1.3294	0.3253	−0.0421	0.120*	
H1C	1.3628	0.3764	0.0213	0.120*	
C2	1.18730 (15)	0.26419 (15)	0.07217 (8)	0.0535 (4)	
C3	1.11793 (16)	0.17477 (14)	0.09004 (8)	0.0543 (4)	
H3	1.1202	0.1066	0.0697	0.065*	
C4	1.04518 (15)	0.18516 (14)	0.13763 (7)	0.0498 (4)	
H4	0.9989	0.1243	0.1493	0.060*	
C5	1.04133 (13)	0.28631 (13)	0.16787 (7)	0.0440 (3)	
C6	1.11545 (18)	0.37376 (16)	0.15177 (9)	0.0649 (5)	
H6	1.1165	0.4408	0.1732	0.078*	
C7	1.18787 (18)	0.36266 (17)	0.10421 (9)	0.0686 (5)	
H7	1.2374	0.4222	0.0937	0.082*	
C8	0.86477 (13)	0.22907 (12)	0.23618 (7)	0.0399 (3)	
H8	0.8950	0.1555	0.2504	0.048*	
C9	0.85697 (14)	0.31425 (13)	0.28832 (7)	0.0445 (4)	
H9	0.7791	0.3557	0.2851	0.053*	
C10	0.95829 (15)	0.37874 (14)	0.25890 (7)	0.0487 (4)	
C11	0.88911 (15)	0.27730 (16)	0.35126 (8)	0.0547 (4)	
C12	0.9571 (2)	0.1818 (2)	0.36449 (10)	0.0858 (7)	
H12	0.9849	0.1384	0.3337	0.103*	
C13	0.9844 (3)	0.1495 (3)	0.42341 (15)	0.1194 (11)	
H13	1.0275	0.0832	0.4319	0.143*	
C14	0.9481 (3)	0.2149 (4)	0.46853 (14)	0.1249 (13)	
H14	0.9668	0.1935	0.5080	0.150*	
C15	0.8846 (3)	0.3113 (4)	0.45635 (12)	0.1164 (11)	
H15	0.8620	0.3568	0.4875	0.140*	
C16	0.8533 (2)	0.3423 (2)	0.39777 (9)	0.0815 (6)	
H16	0.8079	0.4075	0.3898	0.098*	
C17	0.75450 (13)	0.21783 (11)	0.19174 (7)	0.0373 (3)	
H17	0.7839	0.2009	0.1524	0.045*	
C18	0.65972 (12)	0.12687 (11)	0.20676 (6)	0.0359 (3)	
C19	0.53655 (13)	0.17350 (11)	0.17316 (6)	0.0355 (3)	
C20	0.50045 (14)	0.11538 (13)	0.11319 (7)	0.0428 (3)	
C21	0.54296 (15)	0.18742 (13)	0.06529 (7)	0.0456 (4)	
C22	0.54270 (18)	0.16423 (16)	0.00450 (8)	0.0594 (5)	
H22	0.5123	0.0963	−0.0109	0.071*	
C23	0.5883 (2)	0.24404 (19)	−0.03211 (8)	0.0691 (5)	
H23	0.5888	0.2302	−0.0729	0.083*	
C24	0.6338 (2)	0.34542 (19)	−0.00889 (9)	0.0719 (6)	
H24	0.6644	0.3986	−0.0344	0.086*	
C25	0.63443 (17)	0.36850 (16)	0.05125 (8)	0.0596 (5)	
H25	0.6654	0.4363	0.0666	0.071*	
C26	0.58787 (14)	0.28845 (13)	0.08838 (7)	0.0440 (3)	
C27	0.57408 (13)	0.29586 (11)	0.15455 (6)	0.0390 (3)	
C28	0.62610 (13)	0.12351 (13)	0.27200 (7)	0.0417 (3)	
H28A	0.6111	0.0466	0.2841	0.050*	
H28B	0.6925	0.1543	0.2981	0.050*	
C29	0.50982 (14)	0.19455 (12)	0.27545 (6)	0.0420 (3)	
H29	0.5315	0.2737	0.2827	0.050*	
C30	0.41877 (16)	0.15497 (15)	0.31990 (8)	0.0542 (4)	
H30A	0.4530	0.0933	0.3440	0.065*	
H30B	0.3982	0.2160	0.3461	0.065*	
C31	0.30548 (17)	0.11662 (17)	0.28160 (9)	0.0624 (5)	
H31A	0.2689	0.0507	0.2987	0.075*	
H31B	0.2444	0.1760	0.2777	0.075*	
C32	0.35449 (14)	0.08952 (14)	0.22214 (8)	0.0515 (4)	
H32A	0.3944	0.0165	0.2227	0.062*	
H32B	0.2898	0.0910	0.1903	0.062*	
C33	0.69337 (13)	0.01011 (12)	0.18482 (7)	0.0423 (3)	
C34	0.7789 (2)	−0.08957 (17)	0.10623 (11)	0.0783 (6)	
H34A	0.8259	−0.1353	0.1347	0.117*	
H34B	0.8263	−0.0753	0.0726	0.117*	
H34C	0.7045	−0.1282	0.0932	0.117*	
N1	0.96525 (11)	0.29978 (11)	0.21540 (6)	0.0457 (3)	
N2	0.44375 (11)	0.18163 (10)	0.21577 (5)	0.0389 (3)	
O1	1.01442 (13)	0.46527 (10)	0.26924 (6)	0.0684 (4)	
O2	1.25060 (13)	0.24733 (12)	0.02213 (6)	0.0724 (4)	
O3	0.68707 (9)	0.32126 (8)	0.18767 (5)	0.0418 (2)	
O4	0.48789 (10)	0.37590 (9)	0.16712 (5)	0.0473 (3)	
O5	0.44960 (12)	0.02532 (10)	0.10739 (5)	0.0603 (3)	
O6	0.74874 (11)	0.01595 (9)	0.13371 (5)	0.0533 (3)	
O7	0.67044 (13)	−0.07644 (9)	0.20906 (6)	0.0680 (4)	
H4A	0.436 (2)	0.3390 (19)	0.1904 (10)	0.085 (7)*	

### Atomic displacement parameters (Å^2^)

**Table:**

	*U*^11^	*U*^22^	*U*^33^	*U*^12^	*U*^13^	*U*^23^
C1	0.0753 (13)	0.0779 (15)	0.0903 (15)	−0.0008 (11)	0.0333 (12)	0.0179 (12)
C2	0.0455 (8)	0.0526 (10)	0.0629 (11)	0.0034 (8)	0.0083 (7)	0.0055 (8)
C3	0.0618 (10)	0.0396 (9)	0.0621 (10)	0.0056 (8)	0.0088 (8)	0.0027 (8)
C4	0.0539 (9)	0.0366 (9)	0.0594 (10)	−0.0018 (7)	0.0073 (8)	0.0042 (7)
C5	0.0364 (7)	0.0397 (8)	0.0554 (9)	−0.0031 (6)	0.0007 (6)	−0.0012 (7)
C6	0.0669 (11)	0.0501 (11)	0.0793 (13)	−0.0202 (9)	0.0172 (10)	−0.0124 (9)
C7	0.0626 (11)	0.0613 (12)	0.0841 (14)	−0.0220 (9)	0.0210 (10)	−0.0006 (10)
C8	0.0387 (7)	0.0305 (7)	0.0505 (8)	−0.0043 (6)	0.0024 (6)	−0.0023 (6)
C9	0.0422 (8)	0.0377 (8)	0.0532 (9)	−0.0034 (6)	−0.0006 (6)	−0.0080 (7)
C10	0.0489 (8)	0.0377 (9)	0.0585 (10)	−0.0059 (7)	−0.0036 (7)	−0.0058 (7)
C11	0.0479 (9)	0.0587 (11)	0.0562 (10)	−0.0191 (8)	−0.0065 (7)	−0.0042 (8)
C12	0.0937 (16)	0.0783 (15)	0.0797 (15)	−0.0015 (13)	−0.0366 (12)	0.0039 (12)
C13	0.133 (3)	0.110 (2)	0.105 (2)	−0.0220 (19)	−0.064 (2)	0.0228 (19)
C14	0.120 (3)	0.168 (4)	0.080 (2)	−0.064 (3)	−0.0420 (18)	0.029 (2)
C15	0.113 (2)	0.178 (3)	0.0584 (15)	−0.048 (2)	0.0026 (15)	−0.0205 (18)
C16	0.0835 (14)	0.1023 (18)	0.0588 (12)	−0.0198 (13)	0.0059 (10)	−0.0158 (12)
C17	0.0400 (7)	0.0248 (7)	0.0469 (8)	−0.0032 (6)	0.0016 (6)	−0.0028 (6)
C18	0.0368 (7)	0.0243 (7)	0.0461 (8)	−0.0011 (5)	−0.0013 (6)	−0.0010 (6)
C19	0.0389 (7)	0.0249 (7)	0.0422 (7)	−0.0013 (6)	−0.0019 (6)	−0.0012 (6)
C20	0.0461 (8)	0.0333 (8)	0.0479 (8)	−0.0012 (6)	−0.0048 (6)	−0.0050 (6)
C21	0.0505 (8)	0.0408 (9)	0.0446 (8)	0.0029 (7)	−0.0035 (7)	−0.0006 (7)
C22	0.0741 (12)	0.0545 (11)	0.0488 (10)	0.0040 (9)	−0.0017 (8)	−0.0062 (8)
C23	0.0831 (13)	0.0789 (14)	0.0461 (10)	0.0060 (11)	0.0093 (9)	0.0025 (10)
C24	0.0823 (14)	0.0725 (14)	0.0626 (12)	−0.0050 (11)	0.0165 (10)	0.0165 (10)
C25	0.0670 (11)	0.0507 (10)	0.0614 (11)	−0.0087 (9)	0.0070 (9)	0.0086 (8)
C26	0.0463 (8)	0.0373 (8)	0.0480 (9)	0.0017 (7)	−0.0003 (6)	0.0042 (6)
C27	0.0433 (8)	0.0252 (7)	0.0476 (8)	−0.0016 (6)	−0.0032 (6)	−0.0004 (6)
C28	0.0433 (8)	0.0339 (8)	0.0471 (8)	−0.0039 (6)	−0.0022 (6)	0.0044 (6)
C29	0.0537 (8)	0.0283 (7)	0.0436 (8)	−0.0013 (6)	0.0015 (6)	−0.0033 (6)
C30	0.0636 (10)	0.0460 (10)	0.0543 (10)	0.0081 (8)	0.0138 (8)	0.0006 (8)
C31	0.0552 (10)	0.0572 (11)	0.0771 (13)	−0.0034 (9)	0.0202 (9)	0.0052 (9)
C32	0.0424 (8)	0.0471 (9)	0.0649 (11)	−0.0098 (7)	0.0017 (7)	0.0009 (8)
C33	0.0376 (7)	0.0266 (7)	0.0621 (10)	−0.0005 (6)	−0.0012 (7)	−0.0041 (7)
C34	0.0875 (14)	0.0489 (11)	0.1007 (16)	0.0063 (10)	0.0235 (12)	−0.0293 (11)
N1	0.0413 (6)	0.0365 (7)	0.0594 (8)	−0.0099 (5)	0.0042 (6)	−0.0073 (6)
N2	0.0393 (6)	0.0295 (6)	0.0478 (7)	−0.0005 (5)	0.0012 (5)	−0.0006 (5)
O1	0.0796 (9)	0.0453 (7)	0.0804 (9)	−0.0263 (6)	0.0065 (7)	−0.0161 (6)
O2	0.0740 (8)	0.0664 (9)	0.0806 (9)	0.0013 (7)	0.0326 (7)	0.0062 (7)
O3	0.0457 (5)	0.0239 (5)	0.0543 (6)	−0.0041 (4)	−0.0071 (5)	−0.0014 (4)
O4	0.0530 (6)	0.0279 (5)	0.0610 (7)	0.0058 (5)	0.0035 (5)	0.0030 (5)
O5	0.0784 (8)	0.0415 (7)	0.0596 (7)	−0.0196 (6)	−0.0040 (6)	−0.0111 (5)
O6	0.0607 (7)	0.0328 (6)	0.0672 (7)	0.0030 (5)	0.0108 (6)	−0.0112 (5)
O7	0.0825 (9)	0.0265 (6)	0.0970 (10)	−0.0006 (6)	0.0210 (7)	0.0024 (6)

### Geometric parameters (Å, º)

**Table:**

C1—O2	1.421 (2)	C18—C19	1.5935 (19)
C1—H1A	0.9600	C19—N2	1.4426 (18)
C1—H1B	0.9600	C19—C20	1.540 (2)
C1—H1C	0.9600	C19—C27	1.5743 (19)
C2—C7	1.373 (3)	C20—O5	1.2078 (18)
C2—O2	1.373 (2)	C20—C21	1.474 (2)
C2—C3	1.379 (2)	C21—C26	1.383 (2)
C3—C4	1.379 (2)	C21—C22	1.392 (2)
C3—H3	0.9300	C22—C23	1.370 (3)
C4—C5	1.382 (2)	C22—H22	0.9300
C4—H4	0.9300	C23—C24	1.390 (3)
C5—C6	1.380 (2)	C23—H23	0.9300
C5—N1	1.405 (2)	C24—C25	1.377 (3)
C6—C7	1.377 (3)	C24—H24	0.9300
C6—H6	0.9300	C25—C26	1.384 (2)
C7—H7	0.9300	C25—H25	0.9300
C8—N1	1.4811 (18)	C26—C27	1.506 (2)
C8—C17	1.514 (2)	C27—O4	1.3797 (17)
C8—C9	1.554 (2)	C27—O3	1.4264 (17)
C8—H8	0.9800	C28—C29	1.529 (2)
C9—C11	1.498 (2)	C28—H28A	0.9700
C9—C10	1.531 (2)	C28—H28B	0.9700
C9—H9	0.9800	C29—N2	1.4845 (19)
C10—O1	1.2105 (19)	C29—C30	1.529 (2)
C10—N1	1.360 (2)	C29—H29	0.9800
C11—C12	1.376 (3)	C30—C31	1.525 (3)
C11—C16	1.377 (3)	C30—H30A	0.9700
C12—C13	1.389 (3)	C30—H30B	0.9700
C12—H12	0.9300	C31—C32	1.506 (2)
C13—C14	1.357 (5)	C31—H31A	0.9700
C13—H13	0.9300	C31—H31B	0.9700
C14—C15	1.356 (5)	C32—N2	1.4779 (19)
C14—H14	0.9300	C32—H32A	0.9700
C15—C16	1.385 (4)	C32—H32B	0.9700
C15—H15	0.9300	C33—O7	1.1976 (18)
C16—H16	0.9300	C33—O6	1.3344 (19)
C17—O3	1.4315 (16)	C34—O6	1.445 (2)
C17—C18	1.5483 (19)	C34—H34A	0.9600
C17—H17	0.9800	C34—H34B	0.9600
C18—C33	1.5251 (19)	C34—H34C	0.9600
C18—C28	1.535 (2)	O4—H4A	0.91 (2)
			
O2—C1—H1A	109.5	O5—C20—C19	125.39 (14)
O2—C1—H1B	109.5	C21—C20—C19	107.65 (12)
H1A—C1—H1B	109.5	C26—C21—C22	121.05 (16)
O2—C1—H1C	109.5	C26—C21—C20	110.58 (13)
H1A—C1—H1C	109.5	C22—C21—C20	128.37 (15)
H1B—C1—H1C	109.5	C23—C22—C21	118.34 (17)
C7—C2—O2	124.63 (16)	C23—C22—H22	120.8
C7—C2—C3	119.12 (16)	C21—C22—H22	120.8
O2—C2—C3	116.25 (16)	C22—C23—C24	120.63 (18)
C4—C3—C2	120.82 (16)	C22—C23—H23	119.7
C4—C3—H3	119.6	C24—C23—H23	119.7
C2—C3—H3	119.6	C25—C24—C23	121.15 (18)
C3—C4—C5	119.78 (15)	C25—C24—H24	119.4
C3—C4—H4	120.1	C23—C24—H24	119.4
C5—C4—H4	120.1	C24—C25—C26	118.46 (18)
C6—C5—C4	119.20 (15)	C24—C25—H25	120.8
C6—C5—N1	120.03 (15)	C26—C25—H25	120.8
C4—C5—N1	120.76 (14)	C21—C26—C25	120.37 (15)
C7—C6—C5	120.57 (17)	C21—C26—C27	111.31 (13)
C7—C6—H6	119.7	C25—C26—C27	128.30 (15)
C5—C6—H6	119.7	O4—C27—O3	108.88 (11)
C2—C7—C6	120.35 (17)	O4—C27—C26	110.97 (12)
C2—C7—H7	119.8	O3—C27—C26	112.64 (12)
C6—C7—H7	119.8	O4—C27—C19	112.88 (12)
N1—C8—C17	114.43 (12)	O3—C27—C19	106.74 (11)
N1—C8—C9	87.03 (10)	C26—C27—C19	104.68 (11)
C17—C8—C9	118.12 (12)	C29—C28—C18	106.71 (11)
N1—C8—H8	111.7	C29—C28—H28A	110.4
C17—C8—H8	111.7	C18—C28—H28A	110.4
C9—C8—H8	111.7	C29—C28—H28B	110.4
C11—C9—C10	115.14 (13)	C18—C28—H28B	110.4
C11—C9—C8	119.88 (14)	H28A—C28—H28B	108.6
C10—C9—C8	85.54 (11)	N2—C29—C30	105.11 (12)
C11—C9—H9	111.3	N2—C29—C28	104.45 (11)
C10—C9—H9	111.3	C30—C29—C28	116.18 (13)
C8—C9—H9	111.3	N2—C29—H29	110.2
O1—C10—N1	132.13 (16)	C30—C29—H29	110.2
O1—C10—C9	135.43 (16)	C28—C29—H29	110.2
N1—C10—C9	92.43 (12)	C31—C30—C29	105.13 (13)
C12—C11—C16	118.4 (2)	C31—C30—H30A	110.7
C12—C11—C9	122.18 (18)	C29—C30—H30A	110.7
C16—C11—C9	119.40 (18)	C31—C30—H30B	110.7
C11—C12—C13	120.6 (3)	C29—C30—H30B	110.7
C11—C12—H12	119.7	H30A—C30—H30B	108.8
C13—C12—H12	119.7	C32—C31—C30	103.90 (13)
C14—C13—C12	119.9 (3)	C32—C31—H31A	111.0
C14—C13—H13	120.1	C30—C31—H31A	111.0
C12—C13—H13	120.1	C32—C31—H31B	111.0
C15—C14—C13	120.3 (3)	C30—C31—H31B	111.0
C15—C14—H14	119.9	H31A—C31—H31B	109.0
C13—C14—H14	119.9	N2—C32—C31	101.76 (13)
C14—C15—C16	120.3 (3)	N2—C32—H32A	111.4
C14—C15—H15	119.8	C31—C32—H32A	111.4
C16—C15—H15	119.8	N2—C32—H32B	111.4
C11—C16—C15	120.4 (3)	C31—C32—H32B	111.4
C11—C16—H16	119.8	H32A—C32—H32B	109.3
C15—C16—H16	119.8	O7—C33—O6	123.79 (14)
O3—C17—C8	110.33 (11)	O7—C33—C18	124.86 (15)
O3—C17—C18	105.38 (11)	O6—C33—C18	111.31 (12)
C8—C17—C18	115.45 (12)	O6—C34—H34A	109.5
O3—C17—H17	108.5	O6—C34—H34B	109.5
C8—C17—H17	108.5	H34A—C34—H34B	109.5
C18—C17—H17	108.5	O6—C34—H34C	109.5
C33—C18—C28	111.30 (12)	H34A—C34—H34C	109.5
C33—C18—C17	112.66 (12)	H34B—C34—H34C	109.5
C28—C18—C17	115.66 (11)	C10—N1—C5	133.54 (13)
C33—C18—C19	112.15 (11)	C10—N1—C8	94.93 (12)
C28—C18—C19	102.21 (11)	C5—N1—C8	131.52 (12)
C17—C18—C19	101.99 (10)	C19—N2—C32	120.75 (12)
N2—C19—C20	117.45 (12)	C19—N2—C29	106.64 (11)
N2—C19—C27	108.74 (11)	C32—N2—C29	105.71 (12)
C20—C19—C27	103.73 (11)	C2—O2—C1	116.40 (15)
N2—C19—C18	108.44 (11)	C27—O3—C17	105.84 (10)
C20—C19—C18	114.46 (11)	C27—O4—H4A	104.3 (14)
C27—C19—C18	102.74 (10)	C33—O6—C34	116.85 (14)
O5—C20—C21	126.93 (14)		
			
C7—C2—C3—C4	3.1 (3)	C20—C21—C26—C27	−2.66 (18)
O2—C2—C3—C4	−176.14 (16)	C24—C25—C26—C21	0.6 (3)
C2—C3—C4—C5	0.1 (3)	C24—C25—C26—C27	−177.39 (17)
C3—C4—C5—C6	−3.3 (3)	C21—C26—C27—O4	−111.30 (14)
C3—C4—C5—N1	178.20 (15)	C25—C26—C27—O4	66.8 (2)
C4—C5—C6—C7	3.3 (3)	C21—C26—C27—O3	126.34 (13)
N1—C5—C6—C7	−178.19 (17)	C25—C26—C27—O3	−55.6 (2)
O2—C2—C7—C6	176.06 (18)	C21—C26—C27—C19	10.76 (16)
C3—C2—C7—C6	−3.1 (3)	C25—C26—C27—C19	−171.15 (16)
C5—C6—C7—C2	0.0 (3)	N2—C19—C27—O4	−18.91 (16)
N1—C8—C9—C11	−114.51 (14)	C20—C19—C27—O4	106.82 (13)
C17—C8—C9—C11	129.28 (15)	C18—C19—C27—O4	−133.69 (12)
N1—C8—C9—C10	2.02 (11)	N2—C19—C27—O3	100.66 (12)
C17—C8—C9—C10	−114.20 (14)	C20—C19—C27—O3	−133.60 (12)
C11—C9—C10—O1	−60.1 (3)	C18—C19—C27—O3	−14.12 (14)
C8—C9—C10—O1	178.9 (2)	N2—C19—C27—C26	−139.71 (12)
C11—C9—C10—N1	118.83 (15)	C20—C19—C27—C26	−13.98 (14)
C8—C9—C10—N1	−2.19 (12)	C18—C19—C27—C26	105.51 (12)
C10—C9—C11—C12	−81.4 (2)	C33—C18—C28—C29	−135.13 (12)
C8—C9—C11—C12	18.4 (2)	C17—C18—C28—C29	94.62 (14)
C10—C9—C11—C16	96.5 (2)	C19—C18—C28—C29	−15.24 (14)
C8—C9—C11—C16	−163.73 (16)	C18—C28—C29—N2	30.73 (14)
C16—C11—C12—C13	2.6 (3)	C18—C28—C29—C30	145.98 (13)
C9—C11—C12—C13	−179.5 (2)	N2—C29—C30—C31	2.24 (16)
C11—C12—C13—C14	−2.6 (4)	C28—C29—C30—C31	−112.64 (15)
C12—C13—C14—C15	0.4 (5)	C29—C30—C31—C32	23.24 (18)
C13—C14—C15—C16	1.8 (5)	C30—C31—C32—N2	−39.93 (17)
C12—C11—C16—C15	−0.4 (3)	C28—C18—C33—O7	16.1 (2)
C9—C11—C16—C15	−178.4 (2)	C17—C18—C33—O7	147.87 (15)
C14—C15—C16—C11	−1.8 (4)	C19—C18—C33—O7	−97.74 (18)
N1—C8—C17—O3	−72.08 (15)	C28—C18—C33—O6	−165.88 (12)
C9—C8—C17—O3	28.17 (17)	C17—C18—C33—O6	−34.09 (16)
N1—C8—C17—C18	168.60 (12)	C19—C18—C33—O6	80.30 (14)
C9—C8—C17—C18	−91.14 (15)	O1—C10—N1—C5	2.5 (3)
O3—C17—C18—C33	152.85 (11)	C9—C10—N1—C5	−176.45 (17)
C8—C17—C18—C33	−85.15 (15)	O1—C10—N1—C8	−178.7 (2)
O3—C17—C18—C28	−77.57 (14)	C9—C10—N1—C8	2.30 (12)
C8—C17—C18—C28	44.43 (16)	C6—C5—N1—C10	−12.3 (3)
O3—C17—C18—C19	32.43 (13)	C4—C5—N1—C10	166.26 (17)
C8—C17—C18—C19	154.43 (12)	C6—C5—N1—C8	169.41 (16)
C33—C18—C19—N2	113.55 (13)	C4—C5—N1—C8	−12.1 (2)
C28—C18—C19—N2	−5.74 (13)	C17—C8—N1—C10	117.38 (13)
C17—C18—C19—N2	−125.67 (11)	C9—C8—N1—C10	−2.27 (12)
C33—C18—C19—C20	−19.73 (16)	C17—C8—N1—C5	−63.8 (2)
C28—C18—C19—C20	−139.02 (12)	C9—C8—N1—C5	176.52 (16)
C17—C18—C19—C20	101.05 (13)	C20—C19—N2—C32	36.47 (18)
C33—C18—C19—C27	−131.46 (12)	C27—C19—N2—C32	153.77 (12)
C28—C18—C19—C27	109.25 (11)	C18—C19—N2—C32	−95.22 (14)
C17—C18—C19—C27	−10.67 (13)	C20—C19—N2—C29	156.91 (12)
N2—C19—C20—O5	−49.1 (2)	C27—C19—N2—C29	−85.79 (13)
C27—C19—C20—O5	−169.04 (15)	C18—C19—N2—C29	25.22 (14)
C18—C19—C20—O5	79.82 (19)	C31—C32—N2—C19	163.18 (13)
N2—C19—C20—C21	132.86 (13)	C31—C32—N2—C29	42.29 (15)
C27—C19—C20—C21	12.89 (15)	C30—C29—N2—C19	−157.37 (12)
C18—C19—C20—C21	−98.24 (14)	C28—C29—N2—C19	−34.59 (14)
O5—C20—C21—C26	175.07 (16)	C30—C29—N2—C32	−27.70 (15)
C19—C20—C21—C26	−6.91 (17)	C28—C29—N2—C32	95.08 (13)
O5—C20—C21—C22	−5.5 (3)	C7—C2—O2—C1	−13.7 (3)
C19—C20—C21—C22	172.56 (16)	C3—C2—O2—C1	165.57 (17)
C26—C21—C22—C23	0.1 (3)	O4—C27—O3—C17	158.04 (11)
C20—C21—C22—C23	−179.33 (17)	C26—C27—O3—C17	−78.43 (13)
C21—C22—C23—C24	0.1 (3)	C19—C27—O3—C17	35.91 (14)
C22—C23—C24—C25	0.0 (3)	C8—C17—O3—C27	−168.83 (11)
C23—C24—C25—C26	−0.3 (3)	C18—C17—O3—C27	−43.58 (14)
C22—C21—C26—C25	−0.4 (2)	O7—C33—O6—C34	1.6 (2)
C20—C21—C26—C25	179.08 (15)	C18—C33—O6—C34	−176.43 (14)
C22—C21—C26—C27	177.83 (15)		

### Hydrogen-bond geometry (Å, º)

**Table:**

*D*—H···*A*	*D*—H	H···*A*	*D*···*A*	*D*—H···*A*
O4—H4*A*···N2	0.91 (2)	1.95 (2)	2.6120 (16)	127.8 (19)
C6—H6···O1	0.93	2.51	3.122 (2)	124
C9—H9···O3	0.98	2.38	2.8220 (19)	107
C17—H17···O6	0.98	2.26	2.7277 (17)	108
C28—H28*A*···O7	0.97	2.35	2.8224 (19)	109
C8—H8···O1^i^	0.98	2.52	3.4047 (19)	151
C28—H28*A*···O4^ii^	0.97	2.58	3.5069 (19)	161

Symmetry codes: (i) −*x*+2, *y*−1/2, −*z*+1/2; (ii) −*x*+1, *y*−1/2, −*z*+1/2.
